# Common methodological pitfalls in ICI pneumonitis risk prediction studies

**DOI:** 10.3389/fimmu.2023.1228812

**Published:** 2023-09-25

**Authors:** Yichen K. Chen, Sarah Welsh, Ardon M. Pillay, Benjamin Tannenwald, Kamen Bliznashki, Emmette Hutchison, John A. D. Aston, Carola-Bibiane Schönlieb, James H. F. Rudd, James Jones, Michael Roberts

**Affiliations:** ^1^ Department of Applied Mathematics and Theoretical Physics, University of Cambridge, Cambridge, United Kingdom; ^2^ Department of Surgery, Cambridge University Hospitals, Cambridge, United Kingdom; ^3^ School of Clinical Medicine, University of Cambridge, Cambridge, United Kingdom; ^4^ Digital Health, Oncology R&D, AstraZeneca, Gaithersburg, MD, United States; ^5^ Department of Pure Mathematics and Mathematical Statistics, University of Cambridge, Cambridge, United Kingdom; ^6^ Department of Medicine, University of Cambridge, Cambridge, United Kingdom; ^7^ Department of Oncology, Cambridge University Hospitals, Cambridge, United Kingdom

**Keywords:** immune checkpoint inhibitor induced pneumonitis, review of methodology, risk of bias assessment, study design, statistical analysis

## Abstract

**Background:**

Pneumonitis is one of the most common adverse events induced by the use of immune checkpoint inhibitors (ICI), accounting for a 20% of all ICI-associated deaths. Despite numerous efforts to identify risk factors and develop predictive models, there is no clinically deployed risk prediction model for patient risk stratification or for guiding subsequent monitoring. We believe this is due to systemic suboptimal approaches in study designs and methodologies in the literature. The nature and prevalence of different methodological approaches has not been thoroughly examined in prior systematic reviews.

**Methods:**

The PubMed, medRxiv and bioRxiv databases were used to identify studies that aimed at risk factor discovery and/or risk prediction model development for ICI-induced pneumonitis (ICI pneumonitis). Studies were then analysed to identify common methodological pitfalls and their contribution to the risk of bias, assessed using the QUIPS and PROBAST tools.

**Results:**

There were 51 manuscripts eligible for the review, with Japan-based studies over-represented, being nearly half (24/51) of all papers considered. Only 2/51 studies had a low risk of bias overall. Common bias-inducing practices included unclear diagnostic method or potential misdiagnosis, lack of multiple testing correction, the use of univariate analysis for selecting features for multivariable analysis, discretization of continuous variables, and inappropriate handling of missing values. Results from the risk model development studies were also likely to have been overoptimistic due to lack of holdout sets.

**Conclusions:**

Studies with low risk of bias in their methodology are lacking in the existing literature. High-quality risk factor identification and risk model development studies are urgently required by the community to give the best chance of them progressing into a clinically deployable risk prediction model. Recommendations and alternative approaches for reducing the risk of bias were also discussed to guide future studies.

## 1 Introduction

Immune checkpoint inhibitors (ICIs) have dramatically improved outcomes in cancer treatment in the past decade. Success has been seen in melanoma, lung and kidney cancer, although their use is rapidly expanding to other cancer types (1). In addition to their use in advanced cancer, they are also used in the perioperative settings to reduce risk of cancer recurrence (2). ICIs most commonly block the programmed cell death protein 1 (PD-1) and cytotoxic T-lymphocyte–associated antigen 4 (CTLA-4) pathways, key negative regulators of the anti-tumour immune response. Despite the success of ICIs, their mechanism of action means that they can trigger immune reactions against non-tumour, healthy tissues. These ‘immune-related adverse events’ (irAEs) may necessitate stopping treatment, or adding other drugs such as steroids to dampen the immune reaction; in rare cases, irAEs are severe enough to require hospital treatment or lead to patient death (3). Early recognition and ideally prevention of irAEs are therefore key challenges in oncology practice.

One of the more common irAEs that lead to drug discontinuation is pneumonitis, also known as interstitial lung disease (ILD), an inflammation of the lung tissue. It accounts for 20% of all ICI-associated deaths (4). Patients with ICI induced pneumonitis (ICI pneumonitis) most commonly present with symptoms of dyspnoea and cough (53 and 35 percent, respectively), while approximately one-third of patients are asymptomatic (5). More than half of patients with ICI pneumonitis may also present with another immune-related adverse event, such as colitis, dermatitis, or thyroiditis (5).

ICI pneumonitis can be challenging to distinguish from other pathologies such as pulmonary embolus, infection, heart failure or underlying cancer progression (6). Strategies to identify patients at risk of pneumonitis, and to recognize it early are a clinical priority (6).

Since the introduction of ICIs, many studies have been conducted to discover risk factors and to build risk prediction models. Both systematic and non-systematic reviews have been written to identify possible mechanisms for ICI pneumonitis (7), to summarize risk factors (8) and to recommend management strategies (9). A meta-analysis published in 2022 summarized the odds ratios from 35 studies between 2000 and 2022 and identified several risk factors that have significant pooled effect (**Table 1**) (8). Three studies aimed at building risk prediction models for ICI pneumonitis in human were also published in the same year (10–12). Chao et al. developed a nomogram from a 164-subject dataset; chronic obstructive pulmonary disease (COPD) diagnosis, PD-L1 expression and interleukin 8 (IL-8) levels were included as final predictors for incidence of ICI pneumonitis in non-small cell lung cancer (NSCLC) patients (10). Jia et al. took the nomogram approach as well, with a 209-subject training set, they identified hypertension, ILD emphysema and platelet/lymphocyte ratio (PLR) as model predictors (11). In contrast, Tan et al. trained a deep neural network on 48 subjects to combine pre-ICI imaging and clinical data, which represents the first application of modern machine learning techniques for ICI pneumonitis risk prediction (12).

**Table 1:** Risk factors found to have significant pooled odds ratio (OR) in a meta-analysis conducted by Zhou et al. (8).

	Covariate-adjusted or not	OR	95% CI
**Squamous cell carcinoma**	y	1.13	1.18-1.45
**Previous thoracic radiotherapy**	y	2.07	1.34-3.19
**Pre-existing radiation-induced pneumonitis**	y	3.62	1.53-8.58
**Pre-existing respiratory disease**	y	2.43	1.45-4.07
**Pre-existing interstitial lung disease**	y	5.78	3.08-10.85
**Pre-existing ground glass attenuation**	y	11.48	1.13-116.74
**Pre-existing honeycombing**	y	6.11	2.37-15.79
**Pre-existing pulmonary emphysema**	y	2.72	1.00-7.36
**Use of pembrolizumab**	y	2.89	1.56-5.35
**High PD-L1 expression**	y	3.59	1.23-10.50
**Hypoalbuminemia**	y	0.3	0.14-0.64
**Smoking history**	n	1.39	1.14-1.71
**Neutrophil-lymphocyte ratio**	n	1.04	1.01-1.08
**C-reactive protein**	n	1.08	1.01-1.16

PD-L1, Programmed death-ligand 1.

Despite these efforts, none of the risk factors and risk prediction models have yet translated through to clinical deployment of risk prediction tools. Due to the lack of large-scale high-quality validation studies for the commonly investigated risk factors, the community does not have good evidence to rely on to form a consensual set of risk factors for risk modelling. Findings on individual risk factors are also inconsistent between studies. This inconsistency may be a result of suboptimal methodology, examples include 1) bias in the study populations; 2) difficulty in ICI pneumonitis diagnosis; 3) increased risk of chance findings in small datasets; 4) bias in statistical analysis.

Risk of bias analysis has been reported only in one previous review (8), which, together with other existing reviews, did not provide any detailed assessment on the methodology or the prevalence of bias-prone practices (7, 9, 13–16). Therefore, in the current systematic review, we present a thorough critical appraisal of the methodology of ICI pneumonitis risk factor identification and risk model development studies. Prevalence of bias-prone approaches is quantified as well.

## 2 Materials and methods

### 2.1 Search strategy and selection criteria

Published works and preprints were identified using a Python interface for arXiv (arxiv=1.4.2) and R interfaces for PubMed (RISmed=2.3.0), medRxiv (medrxivr=0.0.5) and bioRxiv (also medrxivr=0.0.5). The databases were searched from 1 January 2000 to 30 September 2022. An initial high-level search was performed for risk factor and risk prediction studies on anti-cancer drug-related pneumonitis. The subsequent search had a narrower scope, focusing on risk factor and risk prediction studies for ICI-related pneumonitis and specifically included individual ICIs in the search terms. The only preprint was removed after confirming that it did not investigate ICI pneumonitis.

A two-stage process was then adopted to identify papers that reported risk factors or risk models for ICI pneumonitis; first, the title and abstract were screened followed by a second screen of the full article text.

#### 2.1.1 Stage I: title and abstract screening

Three reviewers (Y.C., S.W., M.R.) determined the relevance of the studies based on titles and abstracts. Each paper was assessed by two reviewers independently. Conflicts were resolved by consensus between the three reviewers.

The inclusion criteria were: 1. Indication of risk factors, biomarkers and/or predictive models for ICI pneumonitis, including comparison of ICI pneumonitis incidence between different patient subgroups. 2. Reporting of imaging characteristics for ICI pneumonitis

#### 2.1.2 Stage II: full-text screening

Four reviewers (Y.C., S.W., M.R., J.J.) determined the relevance of the studies based on the full text. Each paper was assessed by two reviewers independently, conflicts were resolved by consensus between the four reviewers.

In this review, we included any original study that reported: risk factors, predictive biomarkers and/or models for ICI pneumonitis (including a comparison of ICI pneumonitis incidence between different patient subgroups). The analysis must use statistical tests or predictive modelling for inclusion in our review.

### 2.2 Risk of bias in individual studies

The QUIPS tool (17) was adopted to assess the risk of bias in the risk factor studies. For studies that aim to develop a risk prediction model, the PROBAST (18) method was used to assess the risk of bias. Each study was independently evaluated by two reviewers (Y.C., A.P.), disagreements were resolved by consensus.

### 2.3 Data analysis

The following information was extracted from the papers (**Supplementary Table 1**) (1): outcome of interest; (2) country in which the data were collected; (3) type of cancer treated by ICI; (4) whether the study reported risk factors or a risk prediction model; (5) sample size; (6) statistical tests or models used; (7) data pre-processing, including discretization and handling of missing-values (8) method for validation if a study describes a risk prediction model; (9) whether the code for training the model and the trained model was publicly available (only for studies reporting risk prediction models); and (10) whether imaging features were involved and how they were extracted.

The extracted information was then profiled to evaluate the prevalence of each suboptimal practice.

### 2.4 Keywords in search strategy

#### 2.4.1 Initial search

The initial search looked for the presence of a combination of keywords in the title and abstract of each study. A study was retained if either the title or the abstract contains at least one of: chemotherapy, TKI, tyrosine kinase inhibitor, immune checkpoint inhibitor, immune checkpoint blockade, ICPI, ICI, mTOR, targeted, immune-related; and at least one of: pneumonitis, interstitial lung disease, ILD; as well as one of: biomarker, biomarkers, predictor, predictors, predictive, predict, predicts, prediction, risk factor, risk factors.

#### 2.4.2 ICI-specific search

The secondary search was conducted in the same way but using different search terms. The title or the abstract must contain at least one of: immune checkpoint inhibitor, immune checkpoint blockade, ICPI, ICI, pembrolizumab, nivolumab, cemiplimab, durvalumab, avelumab, atezolizumab, ipilimumab, tremelimumab, immune-related; and at least one of: pneumonitis, interstitial lung disease, ILD; as well as one of: biomarker, biomarkers, predictor, predictors, predictive, predict, predicts, prediction, risk factor, risk factors.

## 3 Results

### 3.1 Study selection

**Figure 1:**
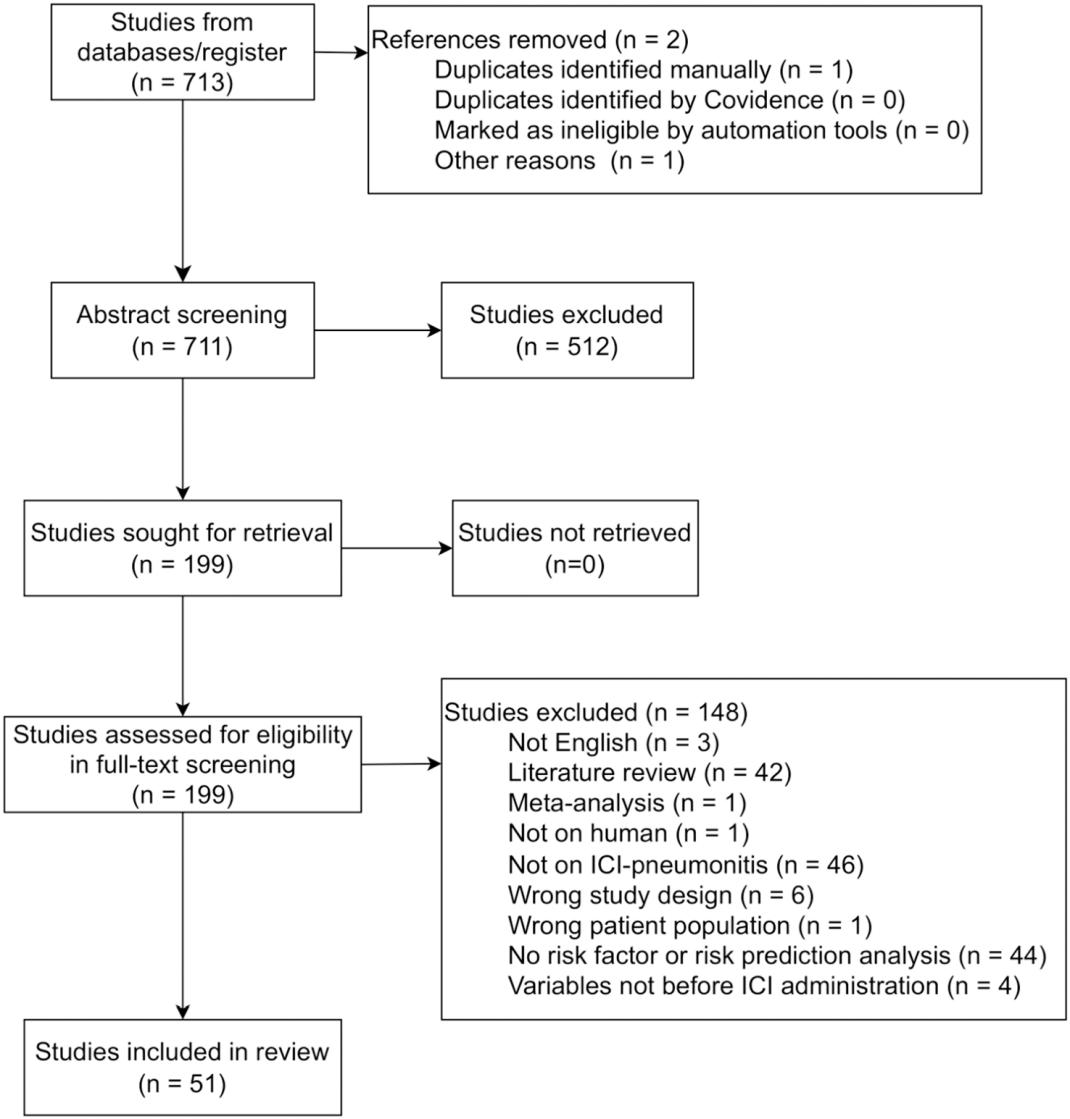
PRISMA flowchart describing the number of studies identified, excluded at abstract and full text screening, and finally included in the analysis.

711 distinct studies were found in the initial search, and 199 of these were deemed relevant during abstract screening. Of the 199 that were eligible for full-text screening, 51 were retained for discussion in this analysis (**Figure 1**, selection criteria are detailed in the Methods section). ICI pneumonitis of different grades were investigated in the studies, including any-grade (48/51), grade 2 or above (3/51), grade 3 or above (3/51), and grade 5 (1/51).

50/51 studies aimed to identify baseline or pre-treatment risk factors for developing ICI pneumonitis from clinical data (46/50), imaging data (27/50, all but 2 included clinical data as well) or specialized laboratory tests such as genetics (2/50) and antibody abundance (2/50). 2/50 studies additionally developed a risk prediction model after risk factor identification from clinical data (10, 11). The remaining study focused on risk prediction model development (with clinical and imaging data) and did not investigate the significance of individual risk factors (12).

#### 3.1.1 Studies investigating risk factors for developing ICI pneumonitis (n=50)

Most (47/50) studies used private data collected from their authors’ affiliated institutions. The remaining 3/51 studies used public databases (Shah 2020 used VigiBase, Asada 2021 and Bai 2021 used data from FDA Adverse Event Reporting System, FAERS), which have limited availability for clinical variables and diagnostic method. Study sample size ranged between 17 and 40826 (19) (median=169, IQR:93-248), with a mean case:control ratio of 21:100 for any-grade (N=47), 16:100 for grade 2 or above (N=3), 11:100 for grade 3 or above (N=3), 14:100 for grade-5 (N=1).

**Figure 2:**
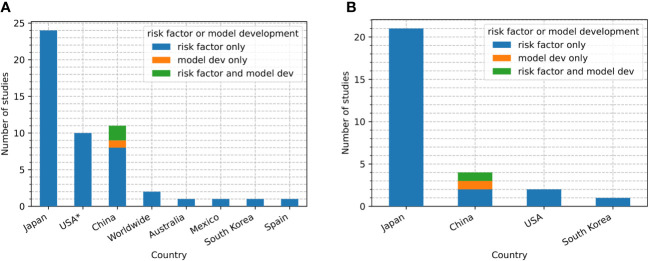
**(A)** Number of studies of each type (risk factor only, risk factor with model development, model development only) conducted in each country. **(B)** Number of studies that used pre-ICI imaging data in each country and each study type (risk factor only, risk factor with model development, model development only). USA: United States. ^✶^One of the ten studies also conducted analysis on data from a multinational database (Shah et al., 2020 (20)).

Close to 50% (24/50) of the studies used data from Japan, followed by USA (10/50), China (10/50), Australia (1/50), South Korea (1/50), Mexico (1/50) and Spain (1/50). 3/51 studies were analysed data from the FAERS database (2/3) and the VigiBase database (1/3) (**Figure 2A**), both contain drug adverse event reports from multiple countries. In addition, 78% (21/27) of studies that examined imaging-based risk factors for ICI pneumonitis development used only Japanese population (**Figure 2B**).

74% (20/27) of imaging-based risk factor studies used computerized tomography (CT) as the sole imaging technique. The remaining 7 did not specify the imaging modality. All the studies relied on manual interpretation to determine radiographic features. 11/27 studies had 2-3 radiologists or pulmonologists reviewing the CT scans, 2/27 studies used a central review committee, 14/27 did not report the number of investigators involved in extracting the imaging findings. Acquisition parameters for the images were described in only 7/27 studies.

**Figure 3:**
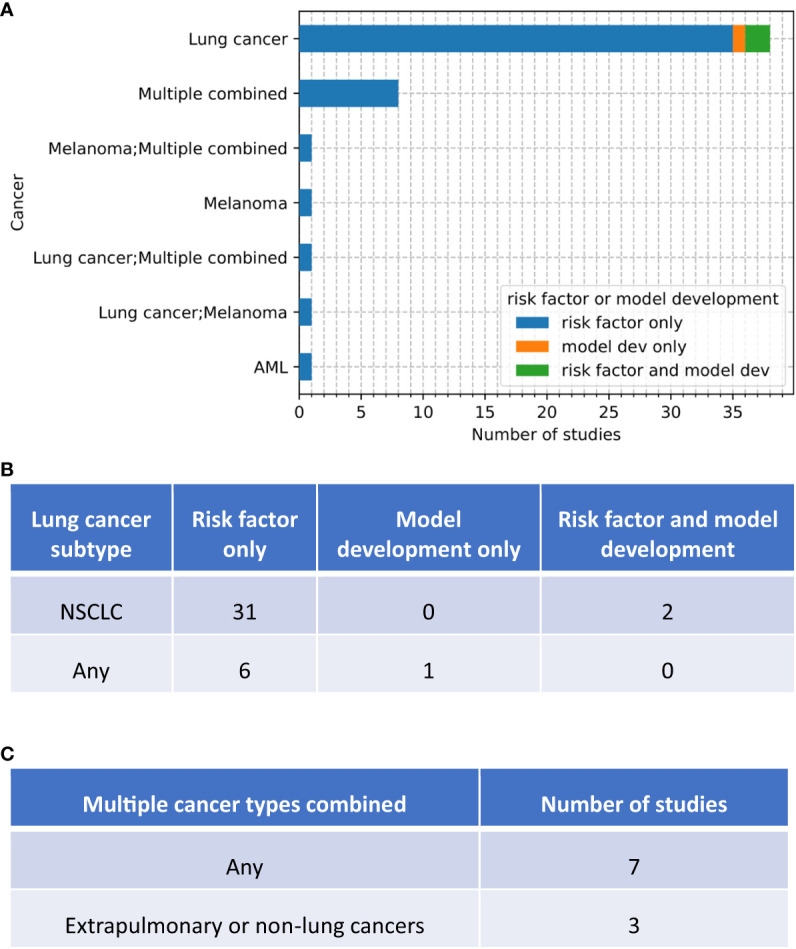
Number of studies of each type (risk factor, risk factor with model development, model development only) that conducted analyses on different types of cancers. **(A)** high-level grouping. If a study conducted two analyses each for a different cancer type, the two cancer types are separated by a semicolon. **(B)** breakdown of lung cancer studies by cancer subtypes. **(C)** break down of combined-cancer studies by inclusion of lung cancer.

A majority of risk factor studies (39/50) conducted analyses on lung cancer, of which 33/38 recruited only NSCLC patients (**Figures 3A, B**). Melanoma was investigated in 3/50 studies. Acute myeloid leukaemia (AML) was considered in one study (**Figure 3A**). Cancers in different organs were combined in the analyses from 10/50 studies, 3/10 excluded subjects with lung cancer (**Figures 3A, C**). One study (19) used data from the FAERS database which contains all the adverse event reports submitted to FDA regardless of cancer type; but the authors did not report the proportion of different cancer types in the data analysed.

19/50 studies summarized follow-up duration. The median time to onset is shorter than the median follow-up duration in all studies that reported both follow-up time and time to onset.

#### 3.1.2 Studies developing ICI pneumonitis risk prediction models (n=3)

A total of 3 studies attempted to develop risk prediction models (10–12). All of them focused on any-grade ICI pneumonitis and used data collected from the their authors’ affiliated institutions, which were all in China (**Figure 2A**). Sample sizes for model development were 48 (12), 164 (10) and 209 (11). Only one study had an internal validation (holdout) set (i.e. was not used in cross-validation or bootstrapping), the same study is also the only one that utilized an external validation set (11). The internal and external validation sets consisted of 209 and 172 subjects, respectively.

Imaging features were investigated in 2/3 studies. The modality of imaging was described in one study (12) that used a deep neural network to implicitly and automatically extract relevant CT imaging features. The other study used imaging-based diagnoses (pre-existing ILD and emphysema status) as candidate predictors but did not explicitly state the imaging domain (11).

In terms of cancer type, all models were built on data from lung cancer patients (**Figures 3A, B**): NSCLC in Chao 2022 and Jia 2022, lung cancer with different histology in Tan 2022.

### 3.2 Risk of bias

Following the recommendations from the Cochrane Prognosis Methods Group, the Quality In Prognosis Studies (QUIPS) tool (17) was adopted to assess the risk of bias in the risk factor studies. A total of six domains are considered by the QUIPS tool: study participation, study attrition, prognostic factor measurement, outcome measurement, study confounding, and statistical analysis and reporting. In the current analysis, the study attrition domain was considered irrelevant since only five studies were prospective and their primary endpoints were either safety or efficacy rather than ICI pneumonitis development. This means the concept of study completion is ill-defined with respect to ICI pneumonitis onset. In addition, due to the lack of understanding and consensus on potential confounding factors for ICI pneumonitis, the risk of bias due to study confounding was not assessed.

For studies that aim to develop a risk prediction model, the Risk Of Bias Assessment Tool (PROBAST) was used (18). The four domains of focus are: participants, predictors, outcomes and analysis.

#### 3.2.1 Studies investigating risk factors for developing ICI pneumonitis (n=50)

##### 3.2.1.1 Study participation

3/50 studies were determined to have high risk of bias (**Table 2**). The three studies with high risk of bias (19, 20, 36) did not provide any summary statistics on the baseline demographic and clinical characteristics of the participants. The remaining 47/50 studies provided a sufficient description of the source of subjects involved and on the distribution of clinical and demographic characteristics in the population, so were considered to have low risk of bias.

**Table 2:** Risk of bias assessment based on QUIPS for the 51 studies that reported risk factors.

Study	Reference	Uses pre-ICI imaging feature	Study participation	Prognostic factor measurement	Outcome measurement	Statistical analysis and reporting	Highest RoB grading
Fujimoto 2018	(21)	n	low	high	high	high	high
Cui 2018	(22)	n	low	low	moderate	moderate	moderate
Nakahama 2018	(23)	y	low	low	high	moderate	high
Owen 2018	(24)	n	low	low	high	moderate	high
Suresh 2018	(25)	n	low	low	moderate	moderate	moderate
Cho 2018	(26)	y	low	low	low	moderate	moderate
Yamaguchi 2018	(27)	y	low	low	low	moderate	moderate
Sukari 2019	(28)	y	low	low	high	high	high
Dávila-Dupont 2019	(29)	n	low	low	low	high	high
Shibaki 2019	(30)	y	low	low	high	low	high
Duma 2019	(31)	n	low	low	high	low	high
Nakanishi 2019	(32)	y	low	high	low	high	high
Komiya 2019	(33)	y	low	high	high	high	high
Fukihara 2019	(34)	y	low	low	high	moderate	high
Tone 2019	(35)	n	low	low	low	moderate	moderate
Tahir 2019	(36)	n	high	low	high	low	high
Nishiyama 2019	(37)	y	low	low	low	moderate	moderate
Sugano 2020	(38)	y	low	low	high	moderate	high
Suzuki 2020	(39)	n	low	high	low	high	high
Shah 2020	(20)	n	high	low	high	high	high
Li 2020	(40)	n	low	high	moderate	moderate	high
Okada 2020	(41)	y	low	low	moderate	high	high
Ikeda 2020	(42)	y	low	low	low	low	low
Moda 2020	(43)	y	low	low	low	high	high
Chu 2020	(44)	n	low	high	high	moderate	high
Shimoji 2020	(45)	y	low	low	low	moderate	moderate
Isono 2020	(46)	y	low	low	high	high	high
Zhang 2020	(47)	y	low	low	moderate	high	high
Asada 2021	(19)	n	high	high	high	moderate	high
Atchley 2021	(48)	y	low	low	high	high	high
Zhou 2021	(49)	n	low	low	high	moderate	high
Bai 2021	(50)	n	low	low	high	high	high
Lin 2021	(51)	n	low	low	low	moderate	moderate
Yamaguchi 2021	(52)	y	low	low	low	high	high
Yamamoto 2021	(53)	y	low	high	high	high	high
Sierra-Rodero 2021	(54)	n	low	high	moderate	high	high
Ichimura 2021	(55)	y	low	high	high	moderate	high
Reuss 2021	(56)	n	low	low	moderate	low	moderate
Yamaguchi 2022	(57)	y	low	low	low	high	high
Wang 2022	(58)	n	low	high	high	high	high
Chao 2022	(10)	n	low	low	moderate	moderate	moderate
Ohe 2022	(59)	y	low	low	high	low	high
Xu 2022	(60)	n	low	low	high	high	high
Sheshadri 2022	(61)	n	low	low	moderate	high	high
Uchida 2022	(62)	y	low	low	low	moderate	moderate
Uhara 2022	(63)	y	low	low	high	moderate	high
Jia 2022	(11)	y	low	high	high	high	high
Ikeda 2022	(64)	y	low	high	low	moderate	high
Abed 2022	(65)	n	low	low	high	low	high
Lu 2022	(66)	y	low	low	low	low	low

Cells are highlighted in red, amber and green to indicate high, moderate and low risk. RoB: risk of bias.

##### 3.2.1.2 Prognostic factor measurement

37/50 studies were considered to have low risk of bias for prognostic factor measurement, with clear information on data source and data handling (**Table 2**). 13/50 studies were evaluated to have high risk of bias due to unclear description on the source of the prognostic factors (2/13, **Table 3**) (33, 55), use of data-driven discretization that was optimized to maximise discrimination (5/13, **Table 3**) (11, 39, 44, 53, 58), and lack of information on data processing such as the handling of missing data or discretization (5/13, **Table 3**) (11, 19, 21, 39, 64). Additionally, 5/13 studies simply excluded subjects with missing data from the analysis when the proportion of missing values were high (> 10% missing, **Table 3**) (32, 40, 53–55).

**Table 3:** Number of risk factor studies judged to have high, moderate and low risk of bias for prognostic factor (risk factor) measurement for different reasons.

Prognostic factor measurement RoB	Data-driven discretization	Excluded missing values when abundant	Unclear pre-processing	Unclear definition or source of risk factor	Number of studies
	(DDD)	(EXCL)	(PREP)	(RFDEF)	
High (DDD)	2	0	0	0	2
High (DDD;EXCL)	1	1	0	0	1
High (DDD;PREP)	1	0	1	0	1
High (DDD;PREP)	1	0	1	0	1
High (EXCL)	0	3	0	0	3
High (PREP)	0	0	3	0	3
High (RFDEF)	0	0	0	1	1
High (RFDEF;EXCL)	0	1	0	1	1
Low	N/A	N/A	N/A	N/A	37
**Total**	**5**	**5**	**5**	**2**	**50**

Reasons for high and moderate risk are listed in the bracket. If multiple reasons are applicable to the same study, the reasons are separated by semicolon. Cells are highlighted in red, amber and green to indicate high, moderate and low risk. RoB: risk of bias.

##### 3.2.1.3 Outcome measurement

All studies explicitly stated ICI pneumonitis as the outcome of interest, alternative terms used in the studies include: pneumonitis (as a type of immune-related adverse event), immune-related pneumonitis/ILD and exacerbation of interstitial pneumonia after ICI administration. 16/50 studies had sufficient description on ICI pneumonitis diagnosis (although no gold-standard diagnostic test exists) or excluded other possible cause of lung inflammation by design, so were considered to have low risk for bias for outcome measurement (**Table 2**). 9/50 had moderate risk, where the authors attempted to distinguish ICI pneumonitis from alternative diagnoses such as infection, tumour progression and pre-existing lesions, but there remains a risk of mistaking radiation pneumonitis (RP) for ICI pneumonitis due to prior thoracic radiotherapy or a lack of information on thoracic radiotherapy (**Table 4**). The remaining 25/50 studies had high risk of bias, 21 of which did not mention isolating ICI pneumonitis from other causes of lung inflammation, 4 had explicitly included RP and other ILD in the definition of ICI pneumonitis (**Table 4**) (19, 34, 48, 55).

**Table 4:** Number of risk factor studies judged to have high, moderate and low risk of bias for outcome measurement for different reasons.

Outcome measurement RoB	Number of studies
High (explicit inclusion of RP and/or other diagnoses)	4
High (lacks diagnostic detail)	21
Moderate (attempted differential diagnosis, risk of RP)	9
Low	16
Total	50

Reasons for high and moderate risk are listed in the bracket. Cells are highlighted in red, amber and green to indicate high, moderate and low risk. RoB: risk of bias, RP: radiation pneumonitis.

##### 3.2.1.4 Statistical analysis and reporting

8/50 studies were considered to have low risk of bias (**Table 2**). 21/50 had moderate risk due to lack of clarity in some but not all analytical steps (4/21, **Table 5**), the use of univariate analysis to select variables for multivariable analysis (11/21, **Table 5**), and discretization of continuous variables (16/21, **Table 5**). The remaining 21/50 were high risk studies (**Table 5**): 11 applied significance test on over 20 predictors in at least one of the analytical steps but used uncorrected p-value < 0.05 as significance threshold; 6 tested the same factor more than once using different discretization thresholds without appropriate multiple testing adjustment; 7 did not provide enough detail to indicate if there was selective reporting of results.

**Table 5:** Number of risk factor studies judged to have high, moderate and low risk of bias for statistical analysis and reporting for different reasons.

Statistical analysis and reporting RoB	> 20 features/factors but use uncorrected p-value < 0.05 as significance threshold (MULTI)	Tested multiple dichotomization thresholds for the same variable (THRESH)	Possible selective reporting (SELECT)	Univariate-based feature selection before multivariable analysis (UNIMULT)	Discretization before modelling/statistical test (DISC)	Unclear description (UNCLR)	Number of studies
High (MULTI)	1	0	0	0	0	0	1
High (MULTI;THRESH)	1	1	0	0	0	0	1
High (MULTI;DISC)	1	0	0	0	1	0	1
High (MULTI; DISC;THRESH;UNIMULT)	1	1	0	1	1	0	1
High (MULTI; UNIMULT; DISC)	2	0	0	2	2	0	2
High (MULTI; UNIMULT; DISC;THRESH)	1	1	0	1	1	0	1
High (SELECT)	0	0	7	0	0	0	7
High (DISC;THRESH)	0	1	0	0	1	0	1
High (UNIMULT;MULTI; DISC)	4	0	0	4	4	0	4
High (UNIMULT; DISC;THRESH)	0	2	0	2	2	0	2
Moderate (DISC)	N/A	N/A	N/A	0	9	0	9
Moderate (UNCLR)	N/A	N/A	N/A	0	0	1	1
Moderate (UNIMULT;DISC;UNCLR)	N/A	N/A	N/A	1	1	1	1
Moderate (UNIMULT)	N/A	N/A	N/A	2	0	0	2
Moderate (UNIMULT; DISC)	N/A	N/A	N/A	6	6	0	6
Moderate (UNIMULT;UNCLR)	N/A	N/A	N/A	2	0	2	2
Low	N/A	N/A	N/A	N/A	N/A	N/A	8
Total with high risk	11	6	7	10	12	0	21
Total with moderate risk	N/A	N/A	N/A	11	16	4	21
Total in all	11	6	7	21	28	4	50

If multiple reasons are applicable to the same study, the reasons are separated by semicolon. Cells are highlighted in red, amber and green to indicate high, moderate and low risk. RoB: risk of bias. Reasons for high and moderate risk are listed in the brackets.

#### 3.2.2 Studies developing ICI pneumonitis risk prediction models (n=3)

##### 3.2.2.1 Participants

According to PROBAST, all three studies reporting risk prediction models had low risk of bias in terms of participant or study sample selection. They retrospectively included data from cancer patients who were given ICI treatments in hospitals, no bias was identified from the inclusion/exclusion criteria.

##### 3.2.2.2 Predictors

All three studies reporting risk prediction models had low risk of bias introduced by predictors or their assessment. Candidate predictors were all extracted pre-treatment or at baseline without knowledge of outcome data. All the predictors were available in training, internal validation, and the external validation sets when used.

##### 3.2.2.3 Outcome

All three studies had unclear risk of bias for outcome determination due to lack of description on how ICI pneumonitis were distinguished from other alternative diagnoses such as infection and tumour progression.

##### 3.2.2.4 Analysis

All three studies had high risk of bias for data analysis due to low sample size:feature ratio (24 cases and 24 controls for deep learning in Tan 2022) (12), discretization of continuous variables (10, 11), exclusion of missing values when a large proportion of a variable is missing (17% missing in Chao 2022) (10), use of univariate analysis to select predictors (10) and uncorrected optimism in the reported model performance (hyperparameter selection with cross-validation and absence of a holdout set in Tan 2022, univariate feature selection based on full population and lack of a holdout set in Chao 2022) (10, 12).

### 3.3 Data analysis

#### 3.3.1 Missing data and imputation

##### 3.3.1.1 Studies investigating risk factors for developing ICI pneumonitis (n=50)

Missing values were reported in 22/50 studies, the maximum proportion of missing values in a single-variable ranges between 0.2% and 64.7% across the studies (median:17.3%, IQR: 7.5%-36.1%). 4/22 of the studies included the missing values as a separate level in regression analysis (11, 35, 41, 46), 5/22 were unclear about the imputation method used (19, 21, 39, 61, 64), the rest simply excluded subjects with missing values. None of the studies investigated whether the missing values were independent from values in other variables.

##### 3.3.1.2 Studies developing ICI pneumonitis risk prediction models (n=3)

2/3 studies reported missing values and also investigated risk factors. One had a maximum of 17% of values missing in a single variable and excluded samples with missing values from analysis (10); the other included missing values as a separate category during model development but did not mention the frequency of missingness (11). Similar to the risk factors studies, the relationship between the missing values and other variables (except for ICI pneumonitis status) were not examined.

#### 3.3.2 Univariate and multivariable analysis

##### 3.3.2.1 Studies investigating risk factors for developing ICI pneumonitis (n=50)

Univariate analysis was performed in 47/50 studies, most of them (26/47) used logistic regression in combination with other tests or by itself: 6/26 studies used contingency and/or two-sample tests to determine variables that should be further assessed by univariate logistic regression (26, 36, 41, 44, 47, 58). One study used contingency test for categorial factors and logistic regression for continuous factors (50). The remaining 19/26 studies used only logistic regression to identify risk factors.

Survival analysis was the second most popular method for univariate analysis, and was performed in 8/47 studies that conducted univariate analysis: 5/8 studies used survival analysis alone to identify risk factors (2 applied the Fine-Gray test, 3 applied Cox proportional hazard model) (40, 46, 53, 61, 63). One study conducted a contingency test and used a Cox proportional hazard model on the same factor (49), the other two used contingency and/or two-sample tests to select variables that should be further assessed in univariate survival modelling (39, 58).

Amongst the remaining studies that conducted univariate analysis, 12 used nothing but contingency and/or two-sample tests, one applied significance test on area under the receiver operating characteristics curve (ROC) (11), and one used general estimating equation (GEE) (56).

46/47 studies that performed univariate analysis used p=0.05 as significance threshold, one implied a threshold of p=0.001 (59). None, except one study, applied multiple testing correction (24).

Multivariable analysis was performed in 31/51 studies, most of them (23/31) used only logistic regression, another 6/31 used only survival modelling (3 with the Fine-Gray test, 3 with the Cox proportional hazard model) (39, 40, 46, 53, 61, 63). One study implied the existence of multivariable analysis for ICI pneumonitis but did not report corresponding results (50), the other applied both logistic regression and Cox proportional hazard model (58). Overall, in either univariate or multivariable analyses, 31/51 studies used logistic regression, 8/51 studies applied survival modelling.

A total of 21/31 studies performing multivariable analysis relied solely (14/21) or partially (7/21) on univariate analysis for feature preselection, so only variables that were below a small p-value (the p-value thresholds ranged between 0.05 and 0.2) or had sufficiently large effect size were passed to the multivariable analysis. 6/31 studies did not conduct any data-driven pre-selection at all (11, 23, 28, 38, 56, 61). 3/31 were unclear on the method of pre-selection (21, 29, 34). All the studies that were partially dependent on univariate analysis for feature selection had additional pre-specified factors. Feature selection during multivariable modelling was conducted in 4/31 studies via stepwise selection (34, 61), shrinkage (43) and p-value cut-off (48).

##### 3.3.2.2 Studies developing ICI pneumonitis risk prediction models (n=3)

Each of the three studies used different tests for univariate analysis. Chao et al. used logistic regression to select predictors for model building (10); Jia et al. used ROC analysis to determine the best dichotomization threshold for continuous variables before multivariable modelling (11); Tan et al. only used univariate contingency and two-sample tests to compare the baseline characteristics between ICI pneumonitis and control subject (12).

The risk prediction models were all multivariable. 2/3 were based on logistic regression (10, 11), they all investigated risk factors as well. The remaining paper experimented different deep learning approaches (unimodal with either clinical factors or CT images alone, multimodal combining the two, and each of these approaches enhanced by contrastive learning) (12).

## 4 Discussion

### 4.1 Study aims

In this analysis, we found that almost all of papers on ICI pneumonitis prediction focused on statistical significance and effect sizes of risk factors rather than risk model development. While statistical significance and the effect sizes (e.g. odds ratio and hazard ratio) may inform clinicians about the relative risk of ICI pneumonitis development for a patient, a clinical decision made without an estimate of the absolute risk can be suboptimal: a 2-fold increase in relative risk could represent a change of absolute risk from 40% to 80%, or from 5% to 10%. We suggest that future studies should particularly focus on validation of existing risk factors and the development of risk prediction models. Both types of study require a much larger sample size than the existing studies (median = 169) for the findings to be considered reliable enough for clinical application. For example, the Liverpool Lung Project (LLP) lung cancer risk prediction model was developed on 1736 subjects and validated on two large cohorts with sample sizes of 2922 and 7652, respectively (67, 68). Furthermore, since distinguishing low grade from high grade ICI pneumonitis is critical for ICI management decisions and the urgency of treatment, risk factors and models that predict high-grade pneumonitis and their time of onset would be highly valuable. However, due to the rarity of high-grade ICI pneumonitis, single-centre studies may find the sample sizes required to be impractical. Multi-national and multi-centre collaboration may be an attractive option in this case.

### 4.2 Dataset considered

In terms of the datasets considered, 47% of studies (24/51, **Figure 2**) were conducted using data from Japan. The proportion increases to 71% (21/28, **Figure 2**) if just the imaging-based studies are considered. This could be due to historically high incidence rates of drug-related ILD in Japan (69). The over-representation of Japanese patient data may lead to inaccurate risk prediction in other countries and ethnicities as models fail to generalize (70). Studies including non-Japanese populations should therefore be encouraged. Most (39/51) studies had specific focus on lung cancer patients, and we suggest that future studies may also concentrate on discovering and validating risk factors and models in non-lung cancer populations to identify whether any generalise to other cancers.

In studies that investigated imaging-based risk factors, all but one study used imaging features that were derived from manually identified abnormal radiological patterns in the lungs. Although the studies sought agreement between multiple radiologists or pulmonologists, subjective bias may still exist and lead to inaccuracy in the labels due to varying levels of experience and training. Automated tools should be developed to identify the radiological patterns to limit such bias.

### 4.3 Outcome definition

One of the main observations from the risk of bias analysis is the between-study inconsistency in the definition of the ICI pneumonitis population and a lack of description on how ICI pneumonitis was distinguished from other diagnoses. This reflects the lack of gold-standard diagnostic criteria for ICI pneumonitis.

Regardless of the cause, patients experiencing pneumonitis will undergo the same or similar clinical management and differentiating the cause of pneumonitis may not necessarily add value to clinical decision-making during its direct management. However, for cancer patients, accurate identification of the cause of pneumonitis in similar subjects is crucial, as if severe ICI induced pneumonitis is suspected this may lead to discontinuation of an effective treatment.

Further studies should endeavour to accurately establish the cause and type of pneumonitis, and document the associated risk factors, so that the community can build a better understanding of managing this clinical situation

### 4.4 Statistical analysis

The risk of bias analysis also revealed some suboptimal techniques in the statistical analysis of the reviewed studies. One prevalent method was the use of univariate analysis to select variables to include in the multivariable analysis, this was observed in 21/31 studies reporting risk factors from multivariable analysis and 1/3 studies reporting risk model development. With this approach, variables that are only informative after controlling for other variables will be dropped out from the final model (71). This phenomenon was observed in one of the studies in our review: Uchida et al. yielded insignificant univariate result for association between pre-existing ILD and risk of symptomatic ICI pneumonitis, but when adjusted for lung metastasis, the association became statistically significant (62). A better alternative would be a step-wise regression or a sparse regularized model (e.g. Least Absolute Shrinkage and Selection Operator (72)).

The widespread lack of multiple testing correction (in all but one study) was another common source of bias when many potential risk factors were simultaneously tested. The bias was exaggerated when the same factors were tested multiple times with different discretization thresholds. For instance, under the assumption of independence between the risk factors, a study should expect at least one false discovery when more than 20 factors are tested at a significance threshold of p = 0.05. This can lead to optimistic and non-reproducible results. To reduce the Type I error, i.e. when a null hypothesis is rejected when it is actually true, methods such as Benjamini-Hochberg correction and Bonferroni correction could be employed (72).

In studies that report risk model development, the most concerning source of optimism came from ill-defined cross-validation where univariate feature selection and data-driven feature transformation (e.g. feature dichotomization based on univariate ROC analysis) was conducted on the entire population rather than the training set in each round of cross-validation. Similarly, reporting only the cross-validation performance after it has been used in hyperparameter optimisation can also lead to an overestimated model performance. To obtain the least biased estimate on model performance, investigators should ideally use internal and (whenever possible) external holdout sets that have not been exposed to any part of model optimisation.

Many (13/22) of the reviewed studies that contained missing values simply discarded the observations from corresponding analyses. This approach assumes that the missing values are randomly distributed and are not related to the outcome or other potential risk factors; this assumption was not verified in any of the studies. As a result, the studies that had sizable numbers of missing values were likely to contain biased results due to failure to account for informative missing values. Statistical power can also be jeopardized due to the reduction in sample size. Future studies should explain and check assumptions on the distribution of missing values, reasons for missingness, and use appropriate imputation methods before considering excluding observations (73). One way to check the informativeness of missing values is to include them as a separate category in the regression analysis, as done by four studies reviewed in this analysis. However, this approach requires discretizing continuous variables, which itself introduces bias (74), so should be discouraged for continuous variables. In places where missing values have been imputed or excluded, non-linear trends involving continuous variables should be investigated using techniques such as spline regression rather than with discretization (74).

We noted the overwhelming popularity of logistic regression in the risk factor analysis studies (55%, either univariate or multivariable) and in risk model development studies (75%). The results from these studies could be interpreted as identifying the odds ratios for (or predicting the risk of) developing ICI pneumonitis before the last follow-up or death regardless of the timing of the events. This limits clinical utility, as the user would not know whether the intervention should be urgent. Recorded follow-up time ranges from days to months, making survival analysis a better and clinically-actionable alternative.

## 5 Conclusion

Overall, our work highlighted several common methodological pitfalls in ICI pneumonitis risk factor identification and risk model development studies, covering areas from diagnosis of ICI pneumonitis to statistical analysis and risk modelling. The majority of the studies considered here are likely to have reported biased results due to those pitfalls. We also provided recommendations and alternative approaches for reducing the risk of bias. Studies with low risk of bias in all domains are lacking in the existing literature (only 2/51, **Table 2**), high-quality risk factor identification and especially risk model development studies (i.e. predicting absolute rather than relative risk) are urgently required by the community in order to progress into formation of a clinical deployable risk prediction model.

## Data availability statement

The original contributions presented in the study are included in the article/**Supplementary Material**. Further inquiries can be directed to the corresponding author.

## Author contributions

Original idea (SW/MR), paper searching (YC), title/abstract/full-text screen (YC/SW/MR/JJ), data extraction and review of bias (YC/AP), draft manuscript (YC/JR/JJ/MR). All authors contributed to the article and approved the submitted version.
